# Calcifying odontogenic cyst of anterior maxillary: Case report and review

**DOI:** 10.1016/j.ijscr.2021.106267

**Published:** 2021-08-06

**Authors:** Mainassara Chekaraou Samir, Gamra Lamiae, Chami Bassima

**Affiliations:** a Faculty of Dentistry-Rabat, Mohammed V University, Rabat, Morocco; b Hassan Pathological Anatomy Center, Rabat, Morocco

**Keywords:** Calcifying odontogenic cyst, Upper maxillae, Decompression, Enucleation

## Abstract

**Introduction and importance:**

Calcifying odontogenic cyst (COC) is a rare lesion of jawbone. It is classified among development cyst in the new WHO classification of tumors of the head and neck in 2017. It is a rare pathology, which is found more in the upper maxillae, with a predominance in women in the second or third decade. The diagnosis is based on the analysis of clinical, radiological and histological features.

**Case presentation:**

We report the case of a 17-year-old patient referred by his orthodontist following the fortuitous discovery of a mixed radiolucent/radiopaque image in the right jaw ranging from the tooth 11 to the tooth 16, for whom the clinical, radiological examination associated with fine needle aspiration cytology suggested a cystic lesion.

**Clinical discussion:**

Management initially consisted of decompression of the lesion and complete enucleation after nine months. Histopathological examination gave the diagnosis of calcifying odontogenic cyst. The follow-up showed favorable evolution.

## Highlights

• Calcifying odontogenic cyst is a rare lesion of the jaws
• Treatment is based on enucleation; in our case we opted for decompression which gave a good result which can be an alternative in large lesions
• This case deserves better exposure in your journal.

## 1 Introduction

Calcifying odontogenic cyst (COC) or Gorlin cyst has been defined as a simple cyst lined with ameloblastoma-like epithelium containing focal ghost cells. In 1971, the COC gained international recognition when the World Health Organization (WHO) [1] included it in its classification of “Histological Typing of Odontogenic Tumors, Jaw Cysts, and Allied Lesions.” [2].

Its classification is still subject to debate. It was classified in 1992 and 2005 by the WHO in odontogenic tumors. In its latest classification in 2017, COC was classified among the developmental cysts [3], [4]. COC is an uncommon lesion, representing 0.1% of all records and 1.3% of all odontogenic cysts with a predominance at the anterior maxillae in patients of the third decade [5].

Below, we report a case of calcifying odontogenic cyst of the maxillary jaw in which decompression allowed the lesion to be reduced in size and its total enucleation after nine months. This case report has been reported in line with the SCARE Criteria [6].

## 2 Case presentation

A 17-year-old patient was referred by his orthodontist after the fortuitous discovery of mixed radiolucent-radiopaque images in the right anterior maxillary sector.

His medical status was unremarkable, with no drug history and allergy.

The extraoral examination was normal. There was no history of trauma, pain, paresthesia or lymphadenopathy.

On intraoral examination, there was a slight swelling in the area extending from the tooth11 to the tooth16. The swelling was covered by a normal mucosa.

On palpation, at the level of posterior maxillary alveolar process, the swelling was hard and painless. It became fluctuating and slightly tender in the anterior region. The cold tests carried out at all teeth of the lesion's area were positive.

**Fig. 1:**
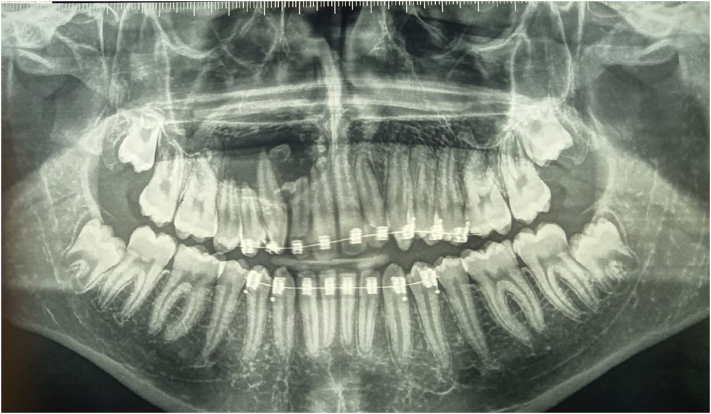
Panoramic radiograph showing a well-defined mixed radiolucent/radiopaque image in the maxillary anterolateral area.

**Fig. 2:**
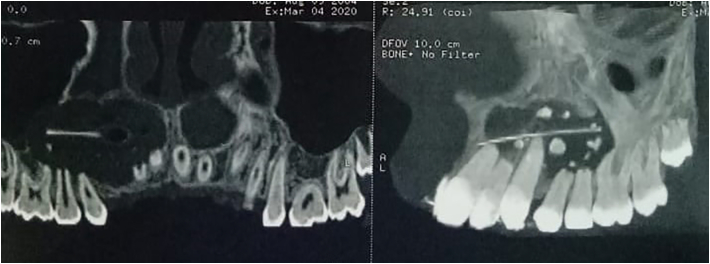
CT scan showing an image of a lytic lesion pushing back the floor of the right maxillary sinus with the presence of hyperdensities in its center.

Digital panoramic radiograph revealed a mixed well-defined radiolucent-radiopaque image located in the area from the tooth 11 to the tooth 16 (Fig. 1), with root resorption of 15, 14, 12 and 11. On computed tomography scan (CT scan) it was noticed an hypodense image (2.7 cm × 2.1 cm) ranging from 11 to 16 with repression of the right maxillary sinus, inside which there are hyperdense images (Fig. 2). CT scan also confirmed the teeth's root resorption.

**Fig. 3:**
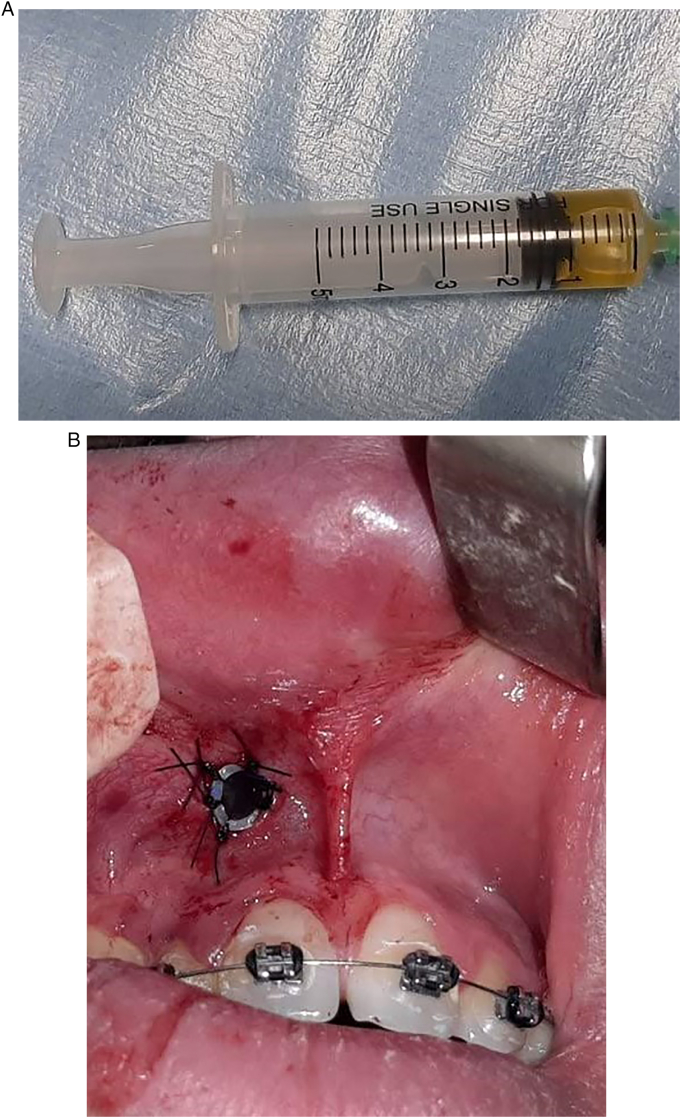
A: Cystic content after aspiration. B: Intraoral view showing the placement of the decompression tube.

Under local anesthesia a cyst decompression was decided. The fine needle aspiration (FNA) of the contents of the lesion brought back a lemon-yellow liquid (3A). Incisional biopsy was performed and a decompression tube was placed (Fig. 3B). Based on the one hand on the history, the clinical and radiographic results and on the other hand on the FNA results, provisional diagnoses were suggested: adenomatoid odontogenic tumor, calcified odontogenic cyst, calcifying epithelial odontogenic tumor (Pindborg tumor) and juvenile cemento-ossifying fibroma in the intermediate stage.

Anatomopathological examination of the specimen gave the diagnosis of cemento-ossifying fibroma.

**Fig. 4:**
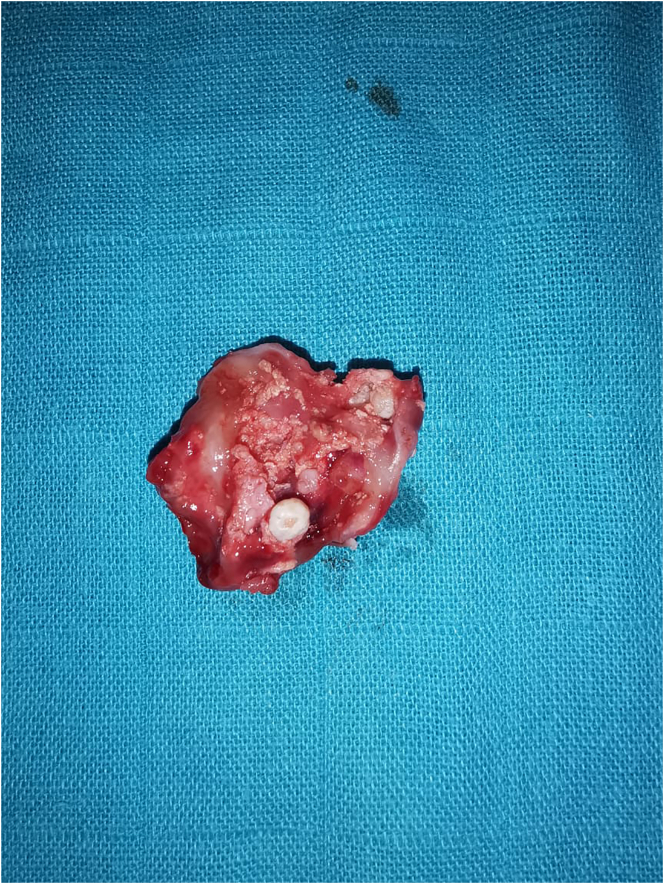
Image of excisional specimen.

9 months later, a reduction in the size of the lesion was observed and a second surgical intervention consisting of a total enucleation of the lesion was performed (Fig. 4). The patient was seen again after 10 days. They are no second effect after the operation.

Histopathological examination of entire lesion showed a cystic wall bordered by an odontogenic epithelium whose basal layer was made of cubocylindrical cells with ameloblastic differentiation. This aspect confirmed the diagnosis of calcifying odontogenic cyst.

**Fig. 5:**
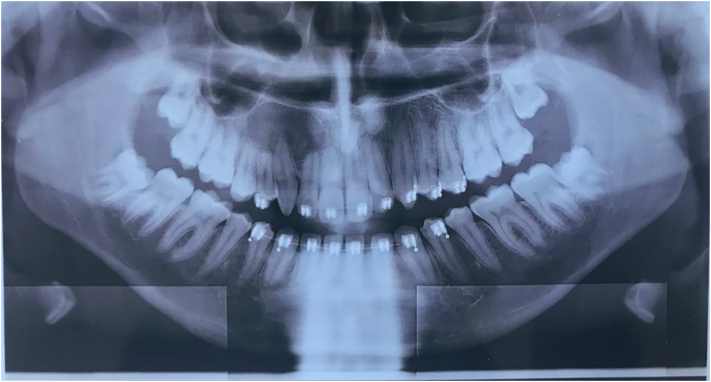
Panoramic radiograph showing the beginning of bone regeneration of the area, 9 months after surgery.

9 months later, the patient underwent an X-ray panoramic control which showed a beginning bone regeneration of the lesion area (Fig. 5).

## 3 Discussion

First described in 1962 by Gorlin, COC is a rare lesion of jaw bone. It is defined as a cyst with an ameloblastoma-like epithelium containing calcifications and focal ghost cells. These cells are also present in dentinogenic ghost cell tumors [5].Because of this diversity, there has been confusion and disagreement in the terminology and classification of this lesion [7]. Some authors considered it as neoplasm, others as cyst.

In 1992, the World Health Organization (WHO) updated the classification of odontogenic tumors and modified COC's name into “calcifying cystic odontogenic tumor” (CCOT) because of their neoplastic behavior [2], [8]. In the latest edition of the WHO classification of tumors of the head and neck (2017), Calcifying odontogenic cyst has been classified among the developmental cysts with two entities: intraosseous and extraosseous [3]. However, Praetorius et al. suggested to classify this lesion as a cyst or tumor (solid). Three different types can be found in the cystic variant: simple unicystic, unicystic odontoma-producing, and unicystic with ameloblastomatous proliferation [9], [10].

According to the study by Arruda et al., COC represents 1.3% of odontogenic cysts, with a distribution of 53% in women and 47% in men [5]. Most patients were adults between (20 to 59 years) accounted for 47.3%, Children and adolescents (0–19 years) accounted for 35.1% of the sample. Its preferred site was the posterior mandibular region, however COCs tend to occur in the anterior portions of both jaws [11].

Swelling in the involved region is for the most common clinical sign noted in patients with COC. Smaller lesions are usually painless and discovered incidentally during routine radiographic evaluation. When located in the maxilla, patients may sometimes complain of nasal stiffness, epistaxis and headache [12].

Radiographically, COC generally appears as a unilocular lesion with a well-defined margin, and contains calcifications as in our case [13]. The frequency of the multilocular form has been noted as 5% and the presence of calcification, which is an important radiographic feature in the interpretation of COC, is detected in about half of all COCs [13]. The frequency of the multilocular form has been noted as 5% and the presence of calcification, which is an important radiographic feature in the interpretation of COC, is detected in about half of all COCs [14].

According to the study of Uchiyama and al, COCs was unilocular, borders were well defined and margins were regular. Root resorption was seen in seven of nine cases and teeth divergence in eight of nine. Impacted teeth were found in six of nine cases; An odontoma was found in one case [15]. On the CT scan, the results were the same as on conventional radiographs; with buccolingual expansion found in 7 of the 9 cases in the sample [15].

The differential diagnosis of COC is made with all mixed lesions of the maxillae. It includes benign radiolucent lesions such as dentigerous cyst, adenomatoid odontogenic tumor, ameloblastic fibro-odontoma, and calcifying epithelial odontogenic tumor [16].

Classical histologic findings in COC are an odontogenic epithelium displaying keratinized ghost cells and calcification. Induction of dental hard tissues may be found in solid COC variants [16].

The recommended treatment for COC consists of enucleation with curettage, which means enucleation followed by removal of a 1- to 2-mm layer of bone around the periphery of the cystic cavity with a sharp curette or a bone bur. [17]. This procedure aims to limit the risk of recurrence. Decompression and marsupialization are conservative treatments used in large lesions with a high success rate as in our case [18].

## 4 Conclusion

Calcified odontogenic cyst is an uncommon benign odontogenic lesion with a higher frequency in the upper maxilla. The treatment is most often conservative. Despite a rare risk of recurrence at 5 years, long term patient follow-up more to 10 years is recommended.

## Ethical approval

Not required.

## Sources of funding

No external funding.

## CRediT authorship contribution statement

**Dr MAINASSARA CHEKARAOU Samir** designed the concept, analyzed and interpreted the findings, wrote and reviewed the final paper under the supervision of Prof **CHAMI Bassima**.

Prof **Lamiae GAMRA** made the lecture of the histological piece.

## Guarantor

Mainassara Samir

## Provenance and peer review

Not commissioned, externally peer-reviewed.

## Consent

Written informed consent was obtained from the patient for publication of this case report and accompanying images. A copy of the written consent is available for review by the Editor-in-Chief of this journal on request.

## Declaration of competing interest

The authors declare no conflict of interest.
